# When music matters: how music app use buffers the relationship between boredom proneness, loneliness and Internet Gaming Disorder

**DOI:** 10.3389/fpsyt.2026.1891113

**Published:** 2026-07-22

**Authors:** Jingyu Yue, Rongxuan Zhai, Zihan Xu, Xudong Wu, Lejun Wang

**Affiliations:** 1 Shanghai Advanced Technical School, Shanghai University of Engineering Science, Shanghai, China; 2 Sport and Health Research Center, Shanghai YangZhi Rehabilitation Hospital (Shanghai Sunshine Rehabilitation Center), Physical Education Department, Tongji University, Shanghai, China; 3 School of Public Administration, Hangzhou Normal University, Hangzhou, China

**Keywords:** boredom proneness, internet gaming disorder, loneliness, music app use, vocational students

## Abstract

**Background:**

Internet Gaming Disorder (IGD) is increasingly prevalent among adolescents and has been associated with psychological factors such as boredom proneness and loneliness. However, the role of everyday digital media use, particularly music app use, in these associations remains unclear. This study aimed to examine whether music app use moderates the associations of boredom proneness and loneliness with IGD risk among secondary vocational school students.

**Methods:**

A cross-sectional study was conducted among 2,341 secondary vocational school students in Shanghai, China, from September to November 2025. Participants completed validated self-report measures assessing demographic characteristics, average daily music app use time, boredom proneness, loneliness, and IGD risk. Pearson correlation analyses were performed to examine bivariate associations. Hierarchical multiple regression analyses were conducted to assess the independent associations of boredom proneness, loneliness, and music app use with IGD risk, as well as the moderating effects of music app use. Simple slopes analyses were performed to interpret significant interactions.

**Results:**

Boredom proneness (r = 0.465, p < 0.001) and loneliness (r = 0.421, p < 0.001) were positively correlated with IGD risk. Hierarchical regression analyses showed that boredom proneness (B = 0.298, p < 0.001), loneliness (B = 0.581, p < 0.001), and music app use time (B = 0.030, p < 0.001) were independently associated with higher IGD risk. A significant interaction effect was found between loneliness and music app use time (B = −0.0027, p < 0.001), whereas the interaction between boredom proneness and music app use was not significant. Simple slopes analyses indicated that the positive association between loneliness and IGD risk was weaker among adolescents with higher levels of music app use.

**Conclusions:**

Boredom proneness and loneliness represent distinct psychological correlates of IGD risk among secondary vocational school students. Music app use may play a differential moderating role in these pathways by attenuating the association between loneliness and IGD risk, but not the association between boredom proneness and IGD risk. These findings highlight the importance of considering specific patterns of digital media use when developing targeted interventions for adolescent IGD prevention.

## 1 Introduction

Internet Gaming Disorder (IGD) has emerged as a growing public health concern among adolescents and young adults worldwide (1, 2). Excessive and maladaptive gaming behaviors have been associated with a range of negative outcomes, including impaired academic functioning, emotional distress, sleep problems, and social difficulties (3, 4). Given its increasing prevalence and associated health burden across different countries and cultural contexts, IGD has attracted considerable attention from researchers and public health professionals worldwide (5). Although IGD risk has been included as a condition warranting further study in the Diagnostic and Statistical Manual of Mental Disorders (DSM-5) and formally recognized in the International Classification of Diseases (ICD-11), its psychological determinants and contextual risk factors remain incompletely understood. Identifying modifiable psychological and behavioral correlates of IGD risk is therefore essential for advancing prevention and early intervention efforts in youth populations.

Accumulating research has increasingly shifted attention from gaming behaviors per se to the underlying psychological processes that may predispose individuals to problematic gaming. Within compensatory internet use frameworks, problematic gaming is often conceptualized as a maladaptive coping strategy through which individuals attempt to manage unmet psychological needs and negative emotional states, such as boredom and loneliness (6). Within the psychological landscape of IGD risk, boredom proneness and loneliness have emerged as prominent empirical focal points. Boredom, defined by low arousal and a perceived lack of meaningful engagement, often compels individuals to pursue the heightened stimulation and immediate reinforcement inherent in online gaming. Empirical evidence suggests that elevated boredom proneness is closely associated with increased gaming frequency and IGD risk (7). Similarly, loneliness, the subjective experience of social deficit, has been identified as a robust predictor of problematic gaming. Online games offer alternative platforms for social interaction and identity exploration, which may be particularly salient for lonely individuals seeking a sense of belonging (8). While both factors are associated with IGD risk, they represent distinct emotional pathways and relate differently to daily digital engagement. Understanding these distinct pathways may help identify contextual factors that strengthen or weaken their associations with IGD risk.

The relevance of these psychological factors may be further shaped by specific educational and developmental contexts. In China, students enrolled in vocational schools represent a distinctive adolescent population that remains relatively understudied in the IGD literature. Vocational education typically emphasizes skill-based training and early career preparation, accompanied by educational structures and daily routines that differ from general academic tracks. During this developmental stage, students are simultaneously navigating identity formation and emotional regulation, while experiencing varying degrees of academic and social demands. Digital media use, including online gaming and music app engagement, constitutes an important component of daily leisure and emotional coping in this group (9). However, despite their potential relevance to behavioral addiction research, vocational school students have been relatively overlooked in empirical studies examining IGD risk and its associated psychological mechanisms (9). This population may be particularly important to study because vocational students often experience distinct educational environments, career-related pressures, and patterns of digital media engagement, all of which may shape their vulnerability to problematic gaming behaviors and related psychological difficulties.

Given that vocational students heavily rely on digital media for emotional coping, it is important to consider which specific digital activities may be particularly relevant to their IGD risk. Within this broader digital media environment, music app use warrants particular consideration. Music app use represents one of the most prevalent and accessible forms of digital media consumption among adolescents. Unlike gaming or social networking, music listening is often embedded in daily routines as a background activity and is frequently used for emotion regulation, mood management, and psychological coping. Social media and short-video platforms are similar to online gaming in that they directly provide social connection, belongingness, and high-intensity stimulation. They compete with gaming for the same compensatory resources. Music listening is different. It neither directly substitutes for unmet social needs nor activates the specific motivational drives that underlie gaming behavior. This non-competing regulatory space makes music app use particularly suitable for examining conditional buffering effects (10). Previous research has shown that moderate music listening may facilitate mood regulation, stress buffering, and psychological recovery, particularly in contexts of loneliness or negative affect (11, 12). However, excessive reliance on music apps has also been associated with behavioral patterns such as heightened preference for immediate reward, experiential avoidance, and increased multitasking-related attentional fragmentation (13). From a cross-media perspective on behavioral addiction, different forms of digital media use may share overlapping psychological and neurobehavioral mechanisms, including enhanced reward system sensitivity, depletion of self-control resources, and reliance on external activities for negative emotion regulation (14). Within the framework of compensatory internet use models, engagement in specific digital activities may therefore shape how individuals cope with unmet psychological needs. Accordingly, music app use may not necessarily represent a direct pathway linking psychological distress to IGD risk, but rather a contextual factor that influences how individuals respond to negative emotional experiences such as loneliness and boredom. Nevertheless, existing studies on IGD risk have largely focused on gaming behaviors, social media use, or overall screen time, while music app use has rarely been examined as a distinct digital behavior or as a potential moderator of key psychological risk factors such as boredom and loneliness. Consequently, little is known about whether music app engagement may strengthen or weaken the associations between these emotional vulnerabilities and IGD risk.

Against this background, the present cross-sectional study aimed to examine the associations between boredom proneness, loneliness scores, music app use intensity, and IGD risk among secondary vocational school students in Shanghai. Specifically, this study sought to (1) investigate the relationships among boredom proneness, loneliness scores, music app use (time), and IGD risk; and (2) test whether music app use intensity moderates the associations between boredom proneness and IGD risk, as well as between loneliness scores and IGD risk. Based on the theoretical and empirical evidence reviewed above, we hypothesized that boredom proneness and loneliness would be positively associated with IGD risk, and that music app use intensity would moderate these associations. By integrating boredom proneness, loneliness, and music app use within a compensatory internet use perspective, and by examining these relationships in an underrepresented educational group, this study seeks to advance understanding of how everyday digital behaviors may shape the associations between emotional vulnerabilities and IGD risk.

## 2 Methods

### 2.1 Overall study design

This study adopted a cross-sectional survey design. Data were collected between September and November 2025 using a multistage cluster sampling strategy. In the first stage, four administrative districts in Shanghai were selected to represent central urban, suburban, and outer suburban areas. From each district, two vocational schools were randomly selected, yielding a total of eight schools. In the second stage, two to three intact classes were randomly selected from each grade (Grades 1–3) within each school, and all students in the selected classes were invited to participate. The survey was administered collectively at the class level. Trained research assistants provided standardized instructions emphasizing anonymity and voluntary participation. Students completed the questionnaire online by scanning a QR code using their personal smartphones. A total of 2,459 questionnaires were distributed across 102 classes from the eight schools. After excluding questionnaires with patterned responses or substantial missing data, 2,341 valid questionnaires were retained, yielding an effective response rate of 95.2%.

Prior to the formal survey, a pilot test with a small sample involving 200 vocational school students was conducted to refine item wording and optimize the response procedure. All scales demonstrated satisfactory internal consistency reliability (Cronbach’s α ≥ 0.80). The study protocol was approved by the Ethics Committee of Tongji University (Approval No. 2020tjdx006). Informed consent was obtained from all participating schools, and individual informed consent was obtained from each student prior to the survey. For participants under 16 years of age, parental or legal guardian consent was also obtained. Participation was voluntary.

### 2.2 Measures

Participants reported their sex, age, grade level. Given that previous research has consistently confirmed the associations of these variables with IGD (15, 16), they were included as covariates in subsequent analyses. Consistent with prior research (17–19), music app use time was measured using a self-report question asking participants to estimate their average daily time spent listening to music via music apps over the past month, in minutes.

IGD risk was assessed using the Chinese version of the Internet Gaming Disorder Scale–Short Form (IGDS9-SF) (20–22), which comprises nine items corresponding to the DSM-5 diagnostic criteria for IGD. Items are rated on a 5-point Likert scale (1 = never to 5 = very often), yielding total scores ranging from 9 to 45, with higher scores indicating greater IGD risk. The scale demonstrated excellent internal consistency in the present sample (Cronbach’s α = 0.89).

Boredom proneness was measured using the eight-item short form of the Multidimensional State Boredom Scale (MSBS-SF) (23). Items are rated on a 7-point Likert scale ranging from 1 (strongly disagree) to 7 (strongly agree), with higher scores indicating greater boredom proneness.

Loneliness scores was assessed using the eight-item short form of the UCLA Loneliness Scale (Version 3) (24, 25),with items rated on a 4-point scale from 1 (never) to 4 (always), and higher total scores reflecting greater perceived loneliness scores. Both scales demonstrated good internal consistency in the current study, with Cronbach’s α values of 0.88 for boredom proneness and 0.84 for loneliness scores.

The Chinese versions of all scales have also been validated and widely used among Chinese adolescents (22–24).

To ensure data quality, standardized instructions were provided to all participants, and anonymity was guaranteed throughout the survey administration. Discriminant validity was further assessed using the heterotrait–monotrait ratio of correlations (HTMT).

### 2.3 Statistical analysis

All statistical analyses were conducted using SPSS 29.0. Descriptive statistics (means, standard deviations, and proportions) were computed for all study variables. All continuous predictors were mean-centered prior to the creation of interaction terms to reduce multicollinearity. Harman’s single-factor test was conducted to evaluate potential common method bias. Normality was assessed using skewness and kurtosis statistics. HTMT values below 0.85 were considered indicative of satisfactory discriminant validity (26). Pearson correlation analyses were performed to examine bivariate associations among music app use time, boredom proneness, loneliness scores and IGD risk. To test the main and interactive effects of psychological factors and music app use (time) on IGD risk, hierarchical multiple regression analyses were conducted. In the first step, demographic covariates (sex, age, grade level) were entered. In the second step, boredom proneness, loneliness scores, and music app use (time) were entered simultaneously to assess their main effects. In the third step, interaction terms between music app use (time) and boredom proneness, as well as between music app use (time) and loneliness scores (music × boredom; music × loneliness), were added to test moderation effects. For significant moderation effects, simple slopes analyses were conducted, and interaction plots were generated to illustrate the predictive effects of psychological factors on Internet gaming disorder risk under low and high music app use conditions. “Low music” and “high music” were defined as one standard deviation below and above the mean of music app use (time), respectively. The plots display the regression slopes along with 95% confidence intervals to provide a clear visualization of the moderation effects (27). Statistical significance was set at p < 0.05.

## 3 Results

### 3.1 Descriptive statistics

Descriptive statistics for all study variables are presented in **Table 1**. Participants reported an average daily music app use of 107.0 minutes (SD = 65.5). Mean scores for boredom proneness, loneliness scores, and IGD risk were 37.36 (SD = 11.03), 20.70 (SD = 4.26), and 32.13 (SD = 9.73), respectively.

**Table 1:** Descriptive statistics (N = 2341, 1102 (47.1%) males).

Variable	M	SD	Min	Max
Age (year)	17.18	2.33	14	20
Music (min)	107.01	65.54	0	389
Boredom proneness	37.36	11.03	8	56
Loneliness scores	20.7	4.26	8	28
IGD risk	32.13	9.73	9	45

### 3.2 Measurement validity and common method bias

Discriminant validity was assessed using the HTMT. HTMT values for each pairwise construct combination were as follows: boredom proneness versus loneliness (HTMT = 0.488), boredom proneness versus IGD risk (HTMT = 0.487), and loneliness versus IGD risk (HTMT = 0.499). All values were below the recommended threshold of 0.85, indicating satisfactory discriminant validity among the study constructs.

Harman’s single-factor test showed that the first unrotated factor accounted for 43.45% of the total variance, which was below the commonly used threshold of 50%, suggesting that common method bias was unlikely to substantially affect the results.

In addition, all study variables demonstrated acceptable levels of skewness (−0.248 to −0.132) and kurtosis (−1.188 to −0.858), indicating no serious violation of the normality assumption.

### 3.3 Correlations

Pearson correlation analyses are summarized in **Table 2**. Music app use (time) was positively associated with boredom proneness (r = 0.052, p = 0.011, negligible effect size), loneliness scores (r = 0.056, p = 0.007, negligible effect size), and IGD risk (r = 0.186, p < 0.001, small effect size). Boredom proneness and loneliness scores were correlated (r = 0.448, p < 0.001, moderate-to-strong effect size). Both boredom proneness (r = 0.465, p < 0.001) and loneliness scores (r = 0.421, p < 0.001, moderate-to-strong effect size) were positively associated with IGD risk.

**Table 2:** Pearson correlation analyses.

Variable	Music app use time (min)	Boredom proneness	Loneliness scores	IGD risk
Music app use time (min)	1	0.052*	0.056*	0.186***
Boredom proneness		1	0.448***	0.465***
Loneliness scores			1	0.421***
IGD risk				1

*p < 0.05, ***p < 0.001.

### 3.4 Hierarchical regression and simple slopes analyses

Hierarchical multiple regression predicting IGD risk is summarized in **Table 3**. The full model explained 30.8% of the variance in IGD risk (R² = 0.308, F = 115.1, p < 0.001).

**Table 3:** Hierarchical regression predicting IGD risk.

Step	Variable	B	95% CI	p	R²	ΔR²	F	ΔF
Step 1					0.025	0.025	14.95	—
	Sex	1.598	[0.817, 2.380]	<0.001				
	Grade	-0.206	[-0.691, 0.280]	0.406				
Step 2					0.3	0.275	142.81	305.51*
	Sex	0.796	[0.131, 1.461]	0.019				
	Grade	-0.218	[-0.629, 0.194]	0.3				
	Boredom proneness	0.298	[0.265, 0.332]	<0.001				
	Loneliness	0.591	[0.504, 0.678]	<0.001				
	Music app use (time)	0.032	[0.022, 0.041]	<0.001				
Step 3					0.308	0.008	115.07	12.88*
	Sex	0.783	[0.121, 1.444]	0.021				
	Grade	-0.203	[-0.613, 0.207]	0.331				
	Boredom proneness	0.298	[0.265, 0.332]	<0.001				
	Loneliness	0.581	[0.495, 0.668]	<0.001				
	Music app use (time)	0.03	[0.021, 0.040]	<0.001				
	Boredom × Music	-0.0002	[-0.001, 0.000]	0.468				
	Loneliness × Music	-0.0027	[-0.004, -0.001]	<0.001				

B, unstandardized regression coefficient; CI, confidence interval; ΔR², change in explained variance; ΔF, F change statistic; *p < 0.001. Continuous predictors were mean-centered before interaction terms were created.

**Figure 1:**
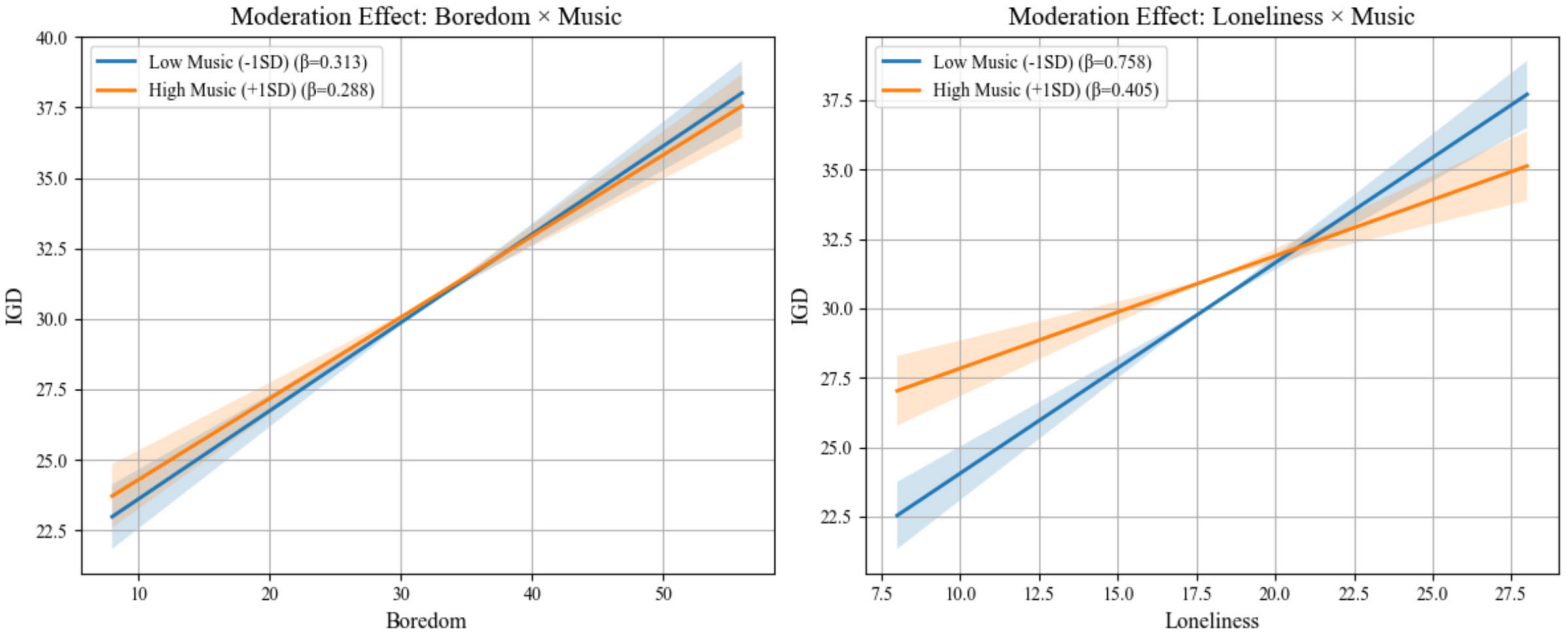
Interaction plot showing the moderating effect of music app use (minutes) on the relationship between loneliness scores and IGD risk. Low and high music conditions are defined as ±1 SD from the mean.

As shown in **Table 3**, boredom proneness [B = 0.298, 95% CI (0.265, 0.332), p < 0.001], loneliness scores [B = 0.581, 95% CI (0.495, 0.668), p < 0.001], and music app use in minutes [B = 0.030, 95% CI (0.021, 0.040), p < 0.001] were positively associated with IGD risk. Music use significantly moderated the association between loneliness scores and IGD risk [B = –0.0027, 95% CI (–0.004, –0.001), p < 0.001], but not boredom proneness [B = –0.0002, 95% CI (–0.001, 0.000), p = 0.468]. The significant interaction between loneliness and music app use is illustrated in **Figure 1**. Simple slopes analyses indicated that the association between loneliness scores and IGD risk varied depending on music app use (time). Specifically, under low music use conditions (-1 SD), the slope was B = 0.758 [95% CI (0.657, 0.861)], whereas under high music use conditions (+1 SD), the slope decreased to B = 0.405 [95% CI (0.301, 0.507)], suggesting that the positive association between loneliness and IGD risk was less pronounced at higher levels of music app use. In contrast, the relationship between boredom proneness and IGD risk did not significantly differ across low [B = 0.313, 95% CI (0.228, 0.394)] and high [B = 0.288, 95% CI (0.203, 0.369)] music use conditions, consistent with the non-significant interaction term.

## 4 Discussion

The present study proposes a psychological–behavioral model of IGD risk in which boredom proneness and loneliness scores represent two distinct emotional correlates, while music app use (time) is examined as a contextual digital moderator. Within this cross-sectional framework, both boredom proneness and loneliness scores are conceptualized as psychological vulnerabilities associated with elevated IGD risk, and everyday engagement with music apps is hypothesized to relate to how these vulnerabilities are associated with problematic gaming. Specifically, the findings indicates that music app use (time) statistically moderates the loneliness–IGD association, such that the positive association between loneliness and IGD risk is weaker at higher levels of music app use, whereas the boredom–IGD association does not show significant moderation. This pattern is consistent with the idea that different negative emotional states may relate to distinct digital coping mechanisms, and that cross-media digital behaviors may have different associations with adolescents’ gaming disorder risk.

Loneliness has consistently been identified as a key psychological correlate of IGD in previous longitudinal research (28), particularly during adolescence, a developmental stage marked by heightened needs for social affiliation and identity validation. According to compensatory internet use models, lonely individuals may engage in online gaming to compensate for unmet offline social needs, as games provide structured social environments offering social presence, belongingness, and rewards (29). Such features may be especially appealing to adolescents experiencing persistent social disconnection (30). The strong association between loneliness scores and IGD risk observed in the present study is consistent with this perspective, supporting the view that problematic gaming often serves a compensatory social function rather than a purely recreational activity. Importantly, the present findings extend this literature by demonstrating that music app use (time) moderates the loneliness–IGD association. Although the interaction coefficient (B = -0.0027) was modest in magnitude, its practical meaning is better understood through the simple slopes it generates: the loneliness–IGD slope decreased by nearly half (from β = 0.758 to β = 0.405) across low to high music use conditions. This suggests that music app use meaningfully attenuates the loneliness–IGD association at the group level, although its effect at the individual level remains modest and should not be overinterpreted as a standalone protective intervention. One plausible interpretation of this moderation is that music listening might serve as an alternative emotion−regulation or social−substitution strategy that partially addresses the affective needs associated with loneliness. Previous research has shown that music can evoke feelings of companionship, emotional resonance, and symbolic social connection even without direct interpersonal interaction (31–33). For lonely adolescents, frequent engagement with music apps may therefore reduce the motivational drive to seek social compensation exclusively through online gaming. As a result, although loneliness scores remains a risk factor for IGD, its translation into problematic gaming behavior appears attenuated under conditions of higher music app use. At a neurobehavioral level, music and gaming may also overlap in their engagement of reward-related and affective processing systems, while differing in their social and cognitive demands (34). Compared with gaming, music listening typically requires lower levels of active involvement, social performance, and sustained cognitive control. This lower-cost regulatory function may allow music use to alleviate negative affect associated with loneliness scores without reinforcing the intensive reward–engagement cycles characteristic of gaming disorder (35). Thus, music app use (time) may represent a relatively lighter compensatory activity that is associated with a reduced, rather than amplified, pathway from loneliness to IGD risk. However, music listening is unlikely to be the only activity associated with reduced loneliness-related gaming risk. Other forms of leisure engagement, physical activity, and social participation may operate through similar mechanisms and warrant further investigation.

In contrast to loneliness scores, boredom proneness showed a robust positive association with IGD risk that was not significantly moderated by music app use (time). This finding suggests that, although correlated, boredom proneness and loneliness scores may be linked to IGD risk through different psychological processes. Boredom proneness is characterized by low arousal, reduced attentional engagement, and a heightened desire for stimulation and novelty (36). From this perspective, individuals high in boredom proneness are motivated to seek activities that provide strong, immediate, and sustained stimulation, a profile that closely matches the structural characteristics of online games (7). Music listening, particularly in everyday app-based formats, may be insufficient to counteract boredom-driven gaming tendencies because it typically offers lower levels of cognitive challenge, interactivity, and reward intensity. While music can modulate mood and provide emotional comfort, it does not substantially increase arousal or satisfy the need for active engagement that underlies boredom proneness. As a result, adolescents experiencing chronic Boredom proneness may continue to seek high-stimulation digital activities, such as gaming, regardless of their level of music app use (37). This pattern could explain why music app use was not found to moderate the boredom–IGD association in the present study. Furthermore, boredom proneness has been linked to deficits in self−regulation and sustained attention, which may increase susceptibility to the immersive and reinforcing nature of games. Unlike loneliness, which might be partially alleviated through affective or symbolic forms of compensation, boredom proneness reflects a motivational state that demands behavioral engagement and novelty (36). Music app use may therefore co−occur with gaming as a parallel or complementary activity rather than acting as a substitute.

To systematically conceptualize the mechanisms underlying the observed moderation effect, we integrate several complementary theoretical perspectives. The Compensatory Internet Use (CIU) model (38) provides the overarching framework, positing that problematic gaming arises from attempts to cope with unmet psychological needs. Self-Determination Theory (39) specifies that loneliness reflects frustration of the relatedness need, motivating individuals to seek social compensation through gaming, whereas music listening serves as an affective regulatory tool that modulates emotional distress without directly satisfying the need itself. The I-PACE model (14) further explains the process through which affective states such as loneliness, combined with reduced self-regulatory capacity, translate into maladaptive gaming behaviors; music app use may alter this process by providing alternative affective resources that reduce compensatory urgency. Dual systems theory (40) adds a developmental lens, suggesting that adolescents’ enhanced reward-seeking system and immature cognitive control system heighten vulnerability to rewarding behaviors like gaming, while music’s lower-intensity rewards offer affective relief without strongly activating the reward system. Finally, media multitasking perspectives (13) help explain why music app use, despite its moderating role for lonely individuals, was positively associated with IGD risk in the full sample—it may reflect a broader pattern of intensive digital engagement rather than a standalone protective factor. Within this integrated multi-level framework, music app use functions as a contextual affective resource that attenuates the loneliness–IGD pathway by reducing the emotional urgency of the compensatory drive, without directly fulfilling the underlying relatedness need or competing with gaming for the same need-satisfying function.

It is worth noting that, although the positive association between loneliness and IGD risk was weaker at higher levels of music app use, music app use itself was slightly positively associated with IGD risk in the full sample. This indicates that, while higher music use may co−occur with a weaker loneliness−IGD link among lonely individuals, extensive engagement with music apps in general may also co−occur with other high−stimulation digital behaviors, including gaming. Therefore, the moderating role of music should be interpreted as conditional: it is associated with a reduced loneliness−related risk but does not act as an overall protective factor against IGD risk.

Taken together, these findings highlight the need to distinguish between different negative emotional states when examining IGD risk, particularly in the context of vocational school students, who face unique schedules, learning demands, and social environments that may be associated with higher boredom proneness and loneliness and may make digital media a readily accessible tool for emotion regulation. From a practical perspective, interventions should be tailored to specific emotional drivers. For lonely students, promoting adaptive music use, defined as intentional, moderate, and context−appropriate music listening for mood regulation rather than excessive or passive consumption, may offer a low−cost emotional resource that attenuates the loneliness–IGD link. For boredom−prone students, however, music listening may be insufficient; these students may benefit more from active, high−engagement activities that provide immediate feedback and sustained stimulation, such as team sports, recreational exercise programs, or outdoor activities, which can offer reward, novelty, and social interaction that compete with the immersive appeal of gaming. This aligns with recent evidence showing that physical activity moderates the association between psychosocial risk factors and gaming disorder among young populations (5). Importantly, such interventions should be context−sensitive, designed to fit the specific schedules, resources, and social dynamics of vocational schools, and emotionally differentiated, targeting loneliness and boredom through distinct strategies rather than a one−size−fits−all approach. For instance, interventions could be delivered through school−based health curricula, after−school sports clubs, or peer−support programs, with a focus on students who exhibit elevated IGD risk or self−reported difficulties with boredom and loneliness. These considerations point to the value of context−sensitive and emotionally differentiated strategies for IGD prevention in vocational school settings.

It is worth noting that the mean IGD risk score in our sample (M = 32.13, SD = 9.73) was higher than that reported in previous studies among adolescents in China (2). In addition, the unstandardized regression coefficient for loneliness (B = 0.581, 95% CI [0.495, 0.668]) was also relatively higher than estimates reported in some general adolescent samples (41). One plausible explanation for both observations relates to the specific characteristics of vocational school students. As noted by Zhai et al. (9), vocational students often face greater behavioral vulnerabilities, including limited self−regulation, reduced familial support, and a higher tendency to use digital devices for emotional coping. These factors may contribute to a stronger association between psychological distress (e.g., boredom proneness, loneliness) and problematic gaming behaviors. Moreover, the same study highlighted that vocational students are more likely to engage in sedentary screen−based activities and report higher levels of digital addiction compared to their peers in general academic tracks. While their study focused on smartphone addiction and musculoskeletal disorders, the underlying mechanism—namely, relying on digital media as a primary emotion−regulation tool in a context of low external support—may similarly explain the elevated IGD risk and the stronger loneliness–IGD association observed in our vocational student sample. Therefore, the higher IGD scores and the relatively strong loneliness association observed in our study likely reflect not only the psychological vulnerabilities we measured but also the broader educational and social context of vocational schooling.

Several limitations of the present study should be acknowledged. First, the cross-sectional design precludes causal inferences regarding the relationships among boredom proneness, loneliness, music app use, and IGD risk. Although the proposed model is theoretically grounded, longitudinal or experimental studies are needed to clarify the temporal ordering and potential reciprocal dynamics among these variables. In particular, the cross-sectional nature of our data cannot establish whether music app use prospectively buffers the loneliness–IGD link or whether individuals with different patterns of IGD risk select into different levels of music engagement over time. Second, all measures were based on self-report questionnaires, which may be subject to recall bias or social desirability effects. Although we employed procedural remedies—including anonymous administration, standardized instructions, and Harman’s single-factor test (which accounted for 43.45% of the total variance, below the 50% threshold)—common method bias cannot be entirely ruled out. Third, and most importantly, music app use was assessed using a single time-based indicator (self-reported daily minutes). While this approach is consistent with prior research and provides a straightforward measure of overall exposure, it does not capture important qualitative dimensions of music engagement, such as listening motives (e.g., emotion regulation, entertainment, or background habit), active versus passive listening, genre preferences, or contextual use patterns (e.g., listening while studying, commuting, or socializing). Consequently, our findings cannot distinguish whether the observed moderation effect is driven by the amount of music listening per se, by the specific functions that music serves for different individuals, or by unmeasured third variables that are associated with both music use and IGD risk. Future studies should adopt more comprehensive measures that capture both the quantity and quality of music engagement, and employ experience sampling methods or objective listening logs to better characterize the role of music in everyday digital coping.

## 5 Conclusion

In this cross−sectional sample of secondary vocational school students, both boredom proneness and loneliness scores were positively associated with IGD risk. Notably, although music app use time was positively associated with IGD risk overall, it moderated the association between loneliness and IGD risk—such that the positive loneliness–IGD link was weaker among participants with higher music app use—whereas it did not significantly moderate the boredom–IGD association. These findings highlight the differential roles of negative emotional states and suggest that preventive interventions may need to address specific emotional drivers. However, causal interpretations are precluded by the cross−sectional design, and future longitudinal research is needed to test whether the observed moderation reflects a genuine buffering process over time.

## Data Availability

The original contributions presented in the study are included in the article/supplementary material. Further inquiries can be directed to the corresponding authors.
